# On oscillatory microstructure during cellular growth of directionally solidified Sn–36at.%Ni peritectic alloy

**DOI:** 10.1038/srep24315

**Published:** 2016-04-12

**Authors:** Peng Peng, Xinzhong Li, Jiangong Li, Yanqing Su, Jingjie Guo

**Affiliations:** 1 Institute of Materials Science and Engineering, Lanzhou University, Lanzhou, China; 2 School of Physical Science and Technology, Lanzhou University, China; 3 School of Materials Science and Engineering, Harbin Institute of Technology, China

## Abstract

An oscillatory microstructure has been observed during deep-cellular growth of directionally solidified Sn–36at.%Ni hyperperitectic alloy containing intermetallic compounds with narrow solubility range. This oscillatory microstructure with a dimension of tens of micrometers has been observed for the first time. The morphology of this wave-like oscillatory structure is similar to secondary dendrite arms, and can be observed only in some local positions of the sample. Through analysis such as successive sectioning of the sample, it can be concluded that this oscillatory microstructure is caused by oscillatory convection of the mushy zone during solidification. And the influence of convection on this oscillatory microstructure was characterized through comparison between experimental and calculations results on the wavelength. Besides, the change in morphology of this oscillatory microstructure has been proved to be caused by peritectic transformation during solidification. Furthermore, the melt concentration increases continuously during solidification of intermetallic compounds with narrow solubility range, which helps formation of this oscillatory microstructure.

An important class of industrial alloys has been represented in peritectic systems, including Fe–Ni, Cu–Zn, Cu–Sn and Ti–Al which are the most widely known^1^,^2^,^3^. Solidification microstructures that form under steady state growth conditions have been reasonably well understood during peritectic solidification. However, under non-steady state growth conditions, the peritectic microstructures is also influenced by many other factors like Gibbs-Thomson effect^4^, and temperature gradient zone melting (TGZM) effect^5^,^6^ etc. Besides, the selection of microstructures in peritectic systems is mostly governed by the coupling effects like convection, nucleation undercooling etc.^7^,^8^.

Due to the above reasons, a wide variety of non-steady state microstructures have been revealed in directional solidification in both the hypoperitectic and hyperperitectic regions of peritectic systems^8^,^9^,^10^,^11^,^12^,^13^,^14^. Among them, the so-called *banded*^9^ structures and the *oscillatory* structures^10^,^12^ have been observed in several peritectic systems, including Sn–Cd, Pb–Bi, Fe–Ni alloys^1^,^3^,^4^,^5^,^6^,^7^,^8^,^9^,^10^,^11^. The former is featured by bands of both the primary and peritectic phases which form alternately normal to the growth direction^9^. And their formations have been attributed to nucleation followed by the growth of one phase (α or β) ahead of a growing planar front of the other phase (β or α). Besides, this *banded* structure is often related to the *oscillatory* structures^10^,^12^. In highly convective hyperperitectic alloys, tree-like structures which appeared as alternate bands and islands in longitudinal cross-sections have been revealed to be continuous structures in Pb–Bi and Sn–Cd alloys^13^,^14^. The *oscillatory* structures are, in fact, a continuous treelike morphology of the primary phase which is surrounded by the peritectic phase^12^. And these structures result from an oscillatory movement of the triple junction due to convection in the melt.

The formation mechanisms of both the *banded* and *oscillatory* structures have been extensively analyzed^8^,^9^,^10^,^11^,^12^,^13^,^14^,^15^,^16^. For the former structure, based on the diffusive model^9^ by Trivedi, the peritectic β phase tends to grow simultaneously in the lateral direction which is normal to the growth of α phase and in the thermal gradient direction in directionally solidified peritectic systems. Thus, the banding cycle which is controlled by the growth competition between the β nuclei and the preexisting α phase under non-steady-state conditions can be observed. The spreading of the peritectic phase on the preexisting primary phase following nucleation was also predicted by phase-field modeling by Lo *et al.*^13^. For the *oscillatory* structure, Park and Trivedi^8^,^12^ have shown that this treelike structure could only be observed in a tube with larger diameter, and disappeared when the diameter of the tube is 0.6 mm or less, suggesting that melt convection has obvious influence on this structure. The model by Karma *et al.*^15^ yields an oscillatory structure which is formed through continuous growth of both phases instead of nucleation at the boundary of the pre-existing phase. Mazumder *et al.*^10^ have further confirmed that oscillating melt convection could lead to an oscillatory, treelike structure. Liu and Trivedi^16^ presented that a large treelike primary phase could be produced by strong oscillatory convection while the banding cycle was independent of it. Thus, the melt concentration should be critical in determining the growth morphology of structures during peritectic solidification^17^,^18^,^19^,^20^,^21^,^22^,^23^.

In the previous analyses discussed above, these non-steady state structures like *banded* and *oscillatory* structures have been confirmed to be significantly influenced by either nucleation competition between α/β phases phase or thermosolutal convection in the melt. However, all of these structures are observed in peritectic systems where both the primary α phase and peritectic β phase are solid solution phases. None of these oscillatory structures have been found in peritectic systems containing intermetallic compounds except that by Kohler *et al.*^3^. For peritectic systems containing intermetallic compounds, as no steady-state solute redistribution of melt can be achieved^24^, some interesting microstructures which are distinct from that of solid solution phase can be obtained^24^,^25^,^26^. In addition, the dimensions of both the *banded* and *band-like* microstructures observed are close to the diameter of the sample. But in our recent experiment, an *oscillatory* microstructure which only exists at few positions of the sample has been observed in directionally solidified Sn–Ni hyperperitectic alloy. The size of this microstructure is much smaller as compared with previous results^8^,^9^,^10^,^11^,^12^,^13^,^14^,^15^,^16^. In a word, this microstructure which is found in peritectic system containing intermetallic compound is distinct from that in peritectic system containing solid solution phases. And this might be attributed to the difference in solute redistribution between intermetallic compounds and solid solution phases.

In this paper we shall present a new kind of *oscillatory* microstructure which has been observed in Sn–Ni peritectic alloy in which both the primary Ni_3_Sn_2_ phase and peritectic Ni_3_Sn_4_ phase are intermetallic compounds with narrow solubility range. Although *banded* or *oscillatory* structure can also be observed at high growth rate^27^,^28^, the growth rate in the present work is rather small. For these *banded* structures^27^,^28^ are formed due to the growth oscillation between planar and cell growth^29^. The aim of this article is threefold: (1) to characterize this *oscillatory* microstructure and analyze how this microstructure is formed; (2) to analyze the influence of convection on formation of this oscillatory microstructure through analyzing the characteristic scale of this structure; (3) to discuss whether the solute redistribution of intermetallic compounds during solidification can influence the morphology of this microstructure.

## Results

**Figure 1:**
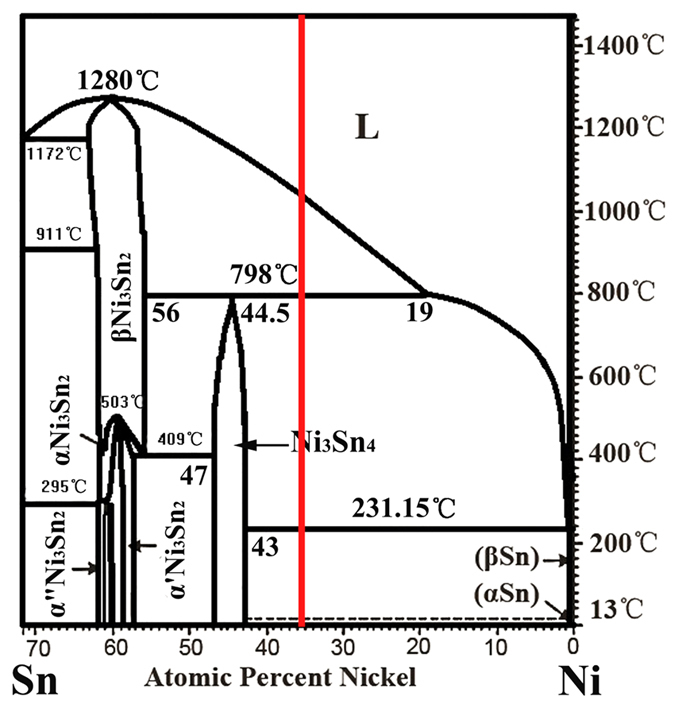
The relevant part of Sn–Ni binary phase diagram^30^.

**Figure 2:**
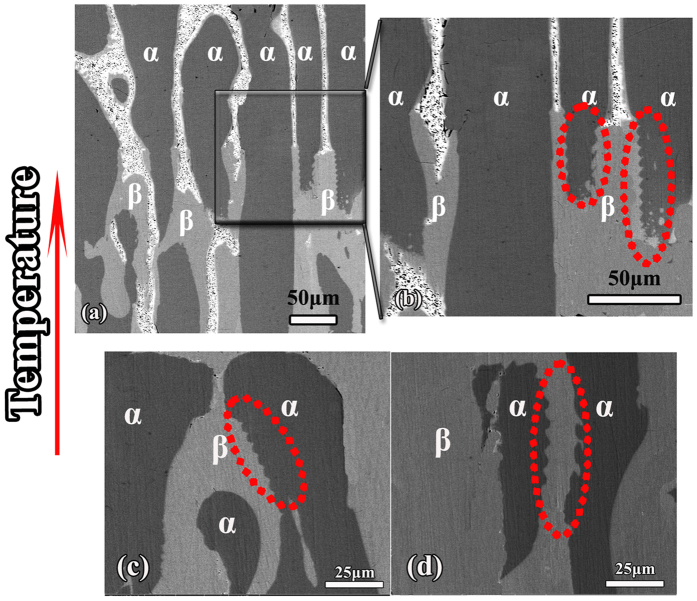
SEM micrographs showing this novel oscillatory microstructure in directionally solidified Sn–36at.%Ni peritectic alloy, and (**b**) is the enlarged view of local position (**a**–**d**) are the views of this novel oscillatory microstructure in the same directionally solidified Sn–36at.%Ni peritectic sample at temperatures lower than those of (**a**,**b**).

Figure 1 shows that under equilibrium solidification^30^, Sn–36at.%Ni alloy^22^ begins at T_L_ = 1040 °C with a primary precipitation of Ni_3_Sn_2_ phase: L→Ni_3_Sn_2_, followed by a peritectic reaction at T_P_ = 798 °C: L + L + Ni_3_Sn_2_→Ni_3_Sn_4_. BSE (backscattered electron) microstructures of the longitudinal sections of directionally solidified Sn–36.at% Ni alloy are shown in Fig. 2. The growth direction of the sample is presented as the arrow direction which is parallel to the temperature gradient. The dark gray/light gray phases represent the primary Ni_3_Sn_2_(α)/peritectic Ni_3_Sn_4_ (β) phases, respectively. The remaining white one represents the eutectic (Ni_3_Sn_4_ + Sn)^4^,^6^,^7^. By relating to the Sn–Ni phase diagram, primary Ni_3_Sn_2_ phase precipitates from the liquid exhibiting deep-cellular morphology. With temperature decreases, the peritectic Ni_3_Sn_4_ phase forms at the primary Ni_3_Sn_2_/liquid interface at T_P_, as illustrated in Fig. 2. It is worth noting that, associated with deep-cellular morphology, an *oscillatory* microstructure with a “wave-like” morphology can only be observed below T_P_, which are marked by the dashed lines. The feature of this morphology is that the interface between Ni_3_Sn_2_/Ni_3_Sn_4_ phases is curved, quite similar to that of secondary branches during dendritic growth. Here (c) and (d) are the views of this novel oscillatory microstructure in the same sample at temperatures lower than those of (a) and (b).

In this work, this *oscillatory* microstructure might be identified as secondary branches or non-steady state *banded* which results from the nucleation competition between Ni_3_Sn_2_ and Ni_3_Sn_4_ phases or continuous *treelike* microstructure^12^ which is caused by solutal convection. The following discussion in this section aims to shed light on how this *oscillatory* microstructure is formed. The first conjecture that the this *oscillatory* microstructure might be identified as secondary branches can be eliminated first. Morphologically speaking, there is an obvious resemblance between this *oscillatory* microstructure and dendrite, which has also been discovered in eutectic alloy^31^. However, this resemblance only shows that they have similar shapes. On the one hand, this *oscillatory* microstructure can only be observed below T_P_ and deep-cellular morphology has been observed above T_P_. On the other hand, the wavelength of this microstructure is much smaller as compared with that of secondary dendrite arms. Thus, this *oscillatory* microstructure is closely associated with peritectic solidification, and can not be identified as stretch of secondary dendrite arms.

The assumption that this *oscillatory* microstructure might be caused by nucleation competition between Ni_3_Sn_2_ and Ni_3_Sn_4_ phases should also be clarified. If the Ni_3_Sn_4_ phase grows laterally, the Ni_3_Sn_2_ phase also advances in the normal direction. This will give rise to a slanted or a curved interface. However, this *oscillatory* microstructure can be observed in only few positions in the sample, indicating that the nucleation competition which occurs in the whole sample can not take place. In addition, if nucleation competition really occurs, then as shown by Gandin *et al.*^32^, the lateral growth of Ni_3_Sn_4_ phase slowly initiates, but after a short time the velocity increases rapidly, approximately as the square of the undercooling. However, abrupt change in the curved Ni_3_Sn_2_/Ni_3_Sn_4_ interface which is caused by this rapid increase of lateral growth of Ni_3_Sn_4_ phase can not be observed. Thus, nucleation competition between Ni_3_Sn_2_ and Ni_3_Sn_4_ phases can also be eliminated.

**Figure 3:**
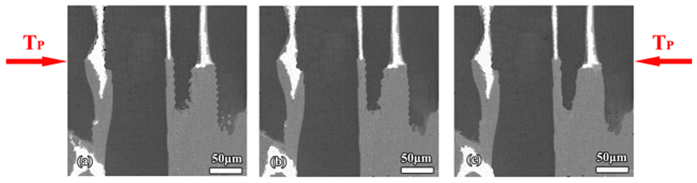
SEM micrographs show the variation of the morphology of this oscillatory after experiencing successive polishing, the polishing depth increases from (**a**–**c**).

Due to the distinct formation mechanism of banded and oscillatory microstructure, it is critical to identify whether this oscillatory-like microstructure in the present work is the *oscillatory* microstructure first. It has been proposed that successive polishing can reveal whether these bands are discrete or interconnected^8^,^12^. Thus, as shown in Fig. 3, successive sectioning of the oscillatory microstructure observed in Fig. 2b has been made. Total polishing height of the sample increases from a to c, and the height of every polishing is 20 μm. It can be deduced from the scales in both Figs 2 and 3 that the dimension of this oscillatory structure is rather small, about 5 ~ 8 μm in average, so the polishing thickness is rather small. Successive sectioning shows that the microstructure does not consist of discrete bands, but is one continuous treelike structure of the peritectic Ni_3_Sn_2_ phase with periodic branches surrounded by the Ni_3_Sn_4_ phase. Besides, it has been confirmed by Trivedi and Park^8^ that fluid flow effects are dominant at low growth rate. Thus, the formation of this oscillatory microstructure can be explained as follows^10^. At the point P where the three phases are in contact and in local equilibrium, solute concentration increases as solidification proceeds. During the solidification of large diameter samples (4 mm) of binary alloys, quasi-periodic convection may set in^10^. The temperature and composition field oscillates in a periodic or quasi-periodic manner, and, hence, the composition profile at the interface may have a complex time-dependent behavior. This, in turn, will cause a periodic or quasi-periodic fluctuating motion of P. And a curve that may look like an oscillating layered structure with arms stretching out is delineated.

## Discussion

As discussed above, this oscillatory microstructure is formed due to oscillatory melt convection. Thus, before analyzing the morphology of this structure, it is necessary to determine the extent of convection during solidification. Dependence of thermosolutal convection on the diameter of the sample has shown that^10^,^15^,^16^ not only the convective transport was greater than diffusive transport, but also the flow was unsteady for tube diameters greater than 3 mm. The flow in the melt for these samples shows periodic or quasi-periodic oscillatory behaviors. Besides, it has been confirmed by Karma *et al.*^15^ that the binary alloy system must be highly convective due to the rejection of lighter solute at the interface, which is similar to Sn–Ni peritectic system.

Mazumder *et al.*^10^ have proposed that the expression *U*_*C*_/*v*_*P*_ could be used to describe the extent of thermosolutal convection: diffusion, steady and unsteady convection. Here *U*_*C*_ is the larger one of fluid rate along/normal to the growth direction; *v*_*P*_ is the growth rate of the sample. According to their numerical analysis, in the case of diffusive transport (*U*_*C*_/*v*_*P*_ ~ 0), the predications by the diffusive model^9^ can be reproduced, and a regular *banded* microstructure can be observed. This is not consistent with the oscillatory microstructure in the present work, indicating that the melt convection can not be neglected. In the case of convection transport, steady/unsteady convection is also distinguished when *U*_*C*_/*v*_*P*_ is 1 ~ 10 or above 100^10^.

Although accurate description of the extent of thermosolutal convection can hardly be obtained through the experimental observation, the range of the extent of thermosolutal convection can be reasonably estimated through experimental observations since different microstructures are formed in the case of different extents of thermosolutal convection. The effect of steady convection is mainly to establish the curvature of the primary/peritectic interface, while the oscillatory interface development between primary and peritectic phases is due to unsteady oscillatory convection^10^. But according to the prediction by Mazumder *et al.*^10^, the fractional area of the respective phases changes with time due to the continuous rejection of solute at the interface and convective-diffusive transport. In the case of steady convection, the fractional area of the primary phase gradually decreases to zero until the interface is completely covered by the peritectic phase. For unsteady oscillatory convection, the fractional area of the primary phase undergoes an oscillatory decay. However, it can be found from Fig. 2 that the oscillatory microstructure in the present work can be formed due to neither steady nor unsteady convection. The decay of the fractional area of the primary phase is not obvious in the present work, and the oscillatory morphology just extends along the curved Ni_3_Sn_2_/Ni_3_Sn_4_ interface. According to Mazumder *et al.*^10^, the decrease rate of fractional area of the primary Ni_3_Sn_2_ phase is about 1.5 times that of the growth velocity. However, the “height” of the wave *h* is defined as the perpendicular distance from the “weak” to the “trough” of the wave. From the experimental results, the values of *h* are about 1.8 ~ 2.5 μm, which is obviously larger than that by steady convection (1.5 μm). Furthermore, the change in wavelength of this oscillatory microstructure is not such obvious. All the observations discussed above show that the extent of thermosolutal convection is in the intermediate state of steady and unsteady convection, that is, 10 < *U*_*C*_/*v*_*P*_ < 100.

**Figure 4:**
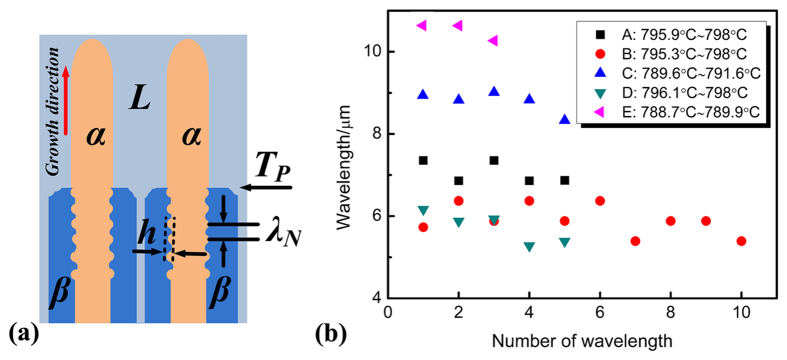
Measurement of the wavelength of this oscillatory microstructure. (**a**) illustration of measurement of the wavelength, (**b**) the variation of wavelength measured with temperature.

Before analyzing the formation of this oscillatory microstructure, it is critical to accurately characterize this wave-like oscillatory structure. The most distinctive feature of this oscillatory microstructure is its wave-like morphology. Thus, the wavelength of this oscillatory microstructure is measured. As shown in Fig. 4a, besides the measured wavelength of this oscillatory microstructure at different locations of the sample, the temperature ranges in which these microstructures exist are also determined. As a matter of fact, different amounts of waves can be observed at the curved Ni_3_Sn_2_/Ni_3_Sn_4_ interface in different deep-cells with oscillatory morphology. The number of wavelength in every oscillatory microstructure is denoted as 1, 2, 3,···N, and the corresponding wavelength measured is λ_1_, λ_2_, λ_3_, ···λ_N_. In the present work, the wavelength is measured from the lower temperature to the higher temperature of the oscillatory microstructure. Thus, the temperature of λ_N_ is higher than that of λ_1_. Oscillatory microstructures having waves less than 4 are neglected for accuracy.

Four features of this oscillatory microstructure can be found from experimental results. The former three ones can be found directly from Fig. 4b. First, the change in wavelength is quite small in the oscillatory microstructure of the same cell. Second, even obvious variation in wavelength of the same cell can not be observed, the wavelengths at lower temperatures are larger than them at higher temperatures in the same cell. Third, in general, the wavelengths at lower temperatures are larger than those at higher temperatures no matter whether they are in the same cell. The last feature which is especially interesting can be found in Fig. 2. That is the curved Ni_3_Sn_2_/Ni_3_Sn_4_ interfaces in this oscillatory microstructure gradually move to the center of the cells during solidification. All of these features suggest that the microstructure change takes place during following solidification, indicating the instability of this oscillatory microstructure.

Based on the discussion in this work, the oscillatory microstructure can be influenced by either oscillatory convection or peritectic transformation. Since peritectic transformation can not change the wavelength of the oscillatory microstructure, it is believed that the extent of the oscillatory convection can be identified if there are changes in the wavelength as a function of distance. It has been proposed that^10^ in the case of oscillatory thermosolutal convection, the wavelengths of both primary and peritectic phases would change with distance. Consequently, the presence of oscillatory convection can be identified if there are changes in the wavelength as a function of distance. Thus, the former three features can be concluded to be caused by convection during solidification, which is distinct from the last feature. Combining with these three features, the effect of convection on this oscillatory microstructure will be analyzed in the Discussion section in detail. In addition, the interesting modification in the curved Ni_3_Sn_2_/Ni_3_Sn_4_ interfaces of this oscillatory microstructure will be discussed as follows first.

It can be observed from Fig. 2 that primary Ni_3_Sn_2_ phase is completely enclosed by peritectic Ni_3_Sn_4_ phase after peritectic reaction. Thus, only solid state transformation can lead to the movement of the curved Ni_3_Sn_2_/Ni_3_Sn_4_ interface of this oscillatory microstructure. During peritectic solidification, the peritectic phase can be formed through three kinds of reactions: peritectic reaction, liquid precipitation and peritectic transformation^1^. Both the solid and liquid phases are involved in the former two reactions while peritectic transformation occurs through solute/solvent transport between primary/peritectic phases. Besides peritectic transformation, coarsening process by the Gibbs-Thomson effect may also lead to change in “wavelength” as that in secondary dendrite arms in peritectic alloys^4^,^5^,^6^.

**Figure 5:**
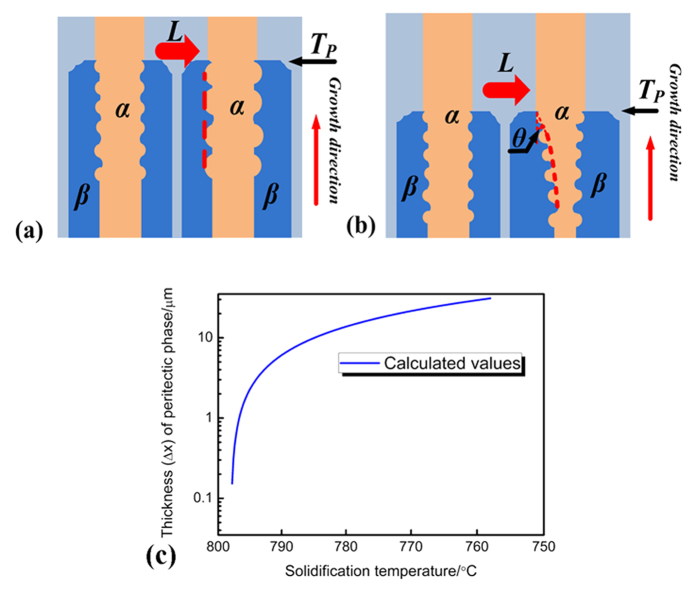
Comparison shows how the primary/peritectic phase interfaces of this oscillatory microstructure are influenced by different solid-state transformation. (**a**) coarsening process and (**b**) peritectic transformation, while (**c**) shows the calculation results on the variation of the thickness *∆x* by peritectic transformation during solidification.

So, the following explanation is presented to distinguish how the curved interfaces of this oscillatory microstructure move. During the coarsening process in solid phases, the fractional area covered by the respective phases will not change with time. As shown in Fig. 5a, a line which interconnects all the troughs of the waves is depicted. If this change is caused by the Gibbs-Thomson effect, as the Ni_3_Sn_2_/Ni_3_Sn_4_ interface keeps still, this line will also keep still. On the contrary, the fractional area covered by the respective phases will change with time if peritectic transformation occurs. Namely, the fractional area by Ni_3_Sn_2_/Ni_3_Sn_4_ phase will decrease/increase. As shown in Fig. 5b, a line which connects all the troughs of the waves is also depicted. During peritectic transformation, the Ni_3_Sn_2_/Ni_3_Sn_4_ interface moves to the center of the cell, and this line also moves to the center of the cell. As the thickness of peritectic phase by peritectic transformation ∆*x* continues increasing during solidification, the Ni_3_Sn_2_/Ni_3_Sn_4_ interface gradually moves to the center of the cell. As a result, an intersection angle *θ* is formed between the initial and final positions of the interfaces.

The latter view that this change in interface is caused by peritectic transformation is supported by our experimental results. The microstructures in Fig. 2 provide an ideal opportunity to find the evidence. A typical deep-cellular growth of primary Ni_3_Sn_2_ phase is illustrated, and the amount of waves is large enough to give an accurate conclusion. Figure 2b shows that the Ni_3_Sn_2_/Ni_3_Sn_4_ interface gradually moves to the center of the cell, and an intersection angle *θ* is formed. Since the rate of peritectic transformation is not constant^33^, the depicted line shown in Fig. 5b is curved. Thus, the intersection angle *θ* is not constant, but changes during solidification. As peritectic transformation has been proved to be the reason for the movement of interfaces, the degree of peritectic transformation is estimated. The description of peritectic transformation has been presented by many researches^33^,^34^,^35^. Here, for the case of simplicity, the analytical presentation by John *et al.*^33^ is used:


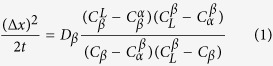


where ∆*x* is the thickness of peritectic phase formed through peritectic transformation. The explanations of these concentrations can be found in Ref. 33. *D*_*β*_ is the average diffusion coefficient of peritectic β phase, and can be expressed as^7^:


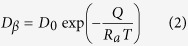


where *D*_0_ is a physical constant depending on the alloy composition, *R*_*a*_ is gas constant, 8.314 J/mol·K., and *Q* is the activation enthalpy, respectively. In Sn–Ni peritectic system, the physical constant *D*_0_ = 5 × 10^−9 ^m^2^/s, the activation enthalpy *Q* = 19150 J/mol is obtained by averaging the values from previous researches^36^,^37^.

The calculation result is illustrated in Fig. 5c. As solidification proceeds, the peritectic phase obtained through peritectic transformation increases very quickly from T_P_ to 788 °C (the lowest temperature at which the oscillatory microstructure is observed). As shown in Fig. 4a, the “height” of the wave *h* is defined as the perpendicular distance from the “weak” to the “trough” of the wave. The values of *h* are influenced by both the oscillatory convection at T_P_ and following peritectic transformation. Since the values of *h* are about 1.8 ~ 2.5 μm, which is much lager than that by the oscillatory convection at T_P_, it therefore can be concluded that the difference in the values of *h* is caused by peritectic transformation which can actually lead to the movement of the Ni_3_Sn_2_/Ni_3_Sn_4_ interface.

**Figure 6:**
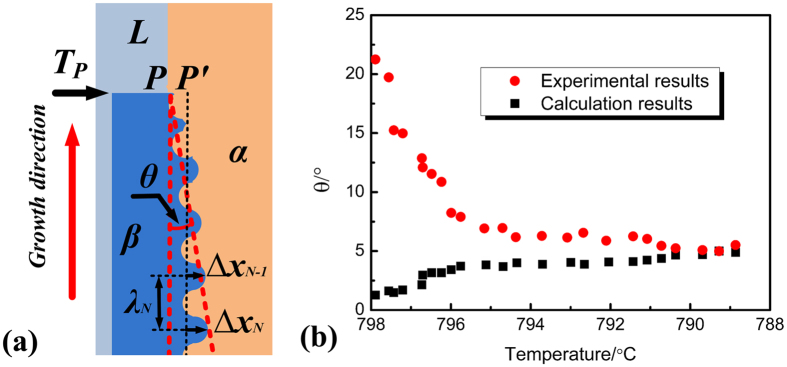
The mechanism how the primary/peritectic phase interface of this oscillatory microstructure move. (**a**) a geometrical relationship between thickness of peritectic phase by peritectic transformation ∆x and λ_N_, (**b**) the comparison between the experimental results and calculation results of the intersection angle *θ* during solidification.

The driving force for peritectic transformation is the diffusion driven by the compositional difference 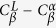
1 across the β phase, causing it to thicken. Here 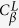 and 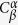 are the concentrations of peritectic β phase in equilibrium with the liquid and primary α phase, respectively. The solubility range of peritectic Ni_3_Sn_4_ phase is much smaller as compared with those of solid solution phases in other peritectic systems. Thus, the driving force for peritectic transformation is small in Sn–Ni peritectic alloy. However, the small thickness of peritectic Ni_3_Sn_4_ phase by peritectic transformation ∆*x* is still large enough to delineate the intersection angle *θ*. For every trough P_N_ (N = 1, 2, 3,···N) at the curved line shown in Fig. 5b, the corresponding wavelength measured is λ_N_, and the corresponding thickness by peritectic transformation is ∆*x*_N_. As shown in Fig. 6a, PP′ is the distance between the peak and of trough of the wave before peritectic transformation. When the number of N is large enough, then, *θ*_N_ is very close to *θ*_N−1_, and a geometrical relationship can be obtained:


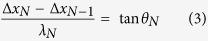


In order to avoid the negative theta, the measurement on the oscillated interfaces including Fig. 2c and d is not carried out along the growth direction of the sample, instead, it is carried out along the curved Ni_3_Sn_2_/Ni_3_Sn_4_ interface.

The experimental results and calculation predictions of the intersection angle *θ* are shown in Fig. 6b. During calculation, the wavelengths λ_N_ used are measured in the experiment. It can be found that the calculation results are always smaller as compared with the experimental results. However, this difference decreases during solidification, and the results are very close to each other at the lowest temperatures. The reason why this difference in intersection angle *θ* occurs maybe found from Equation (3). At temperatures in the vicinity of T_P_, the peritectic phase formed by peritectic transformation is thin, so the values of *θ* obtained through Equation (3) is small. However, in fact, the rationality of Equation (3) lies in that N is large enough, so at the initial of peritectic transformation, this difference is really obvious. Generally speaking, our calculation results overestimate the intersection angle *θ* at every temperature. The most probable reason is that the small thickness of peritectic Ni_3_Sn_4_ phase by peritectic transformation ∆*x* is overestimated. The models on peritectic transformation presented^33^,^34^,^35^ are based on solid solution phases with constant liquidus and solidus slopes. This is distinct from Sn–Ni peritectic system where both the primary Ni_3_Sn_2_ and peritectic Ni_3_Sn_4_ phases are intermetallic compound phases.

In the case of convective flow, the wavelength/band spacing of phases is closely related to the flow in the melt^10^, besides, this characteristic scale can be easily measured. Therefore, it is convenient to analyze the effect of convection on this oscillatory microstructure through comparing the wavelength in the case of purely diffusive and that in the present work. Here, the expressions by Trivedi^9^ are used:


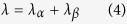



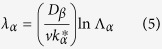



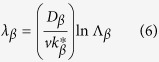



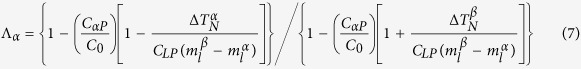



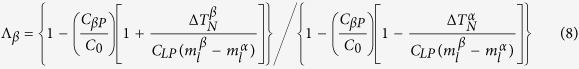


where 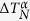 and 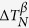 are the undercoolings required for the heterogeneous nucleation of primary and peritectic phases. 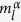 and 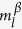
are the liquidus slopes of primary α and peritectic β phases, respectively. *C*_*αP*_, *C*_*βP*_ and *C*_*LP*_ are the solute concentration of α, β and liquid phase in equilibrium at T_P_. 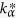 and 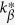 are the modified solute redistribution coefficient which has been proposed by Trivedi and Kurz^25^:


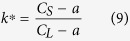


where *a* is the composition at which the linear segments of the solidus and liquidus lines intersect. It can be obtained from Fig. 1 that the values of 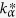 and 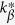 are 0.133 and 0.0893, respectively.

**Figure 7:**
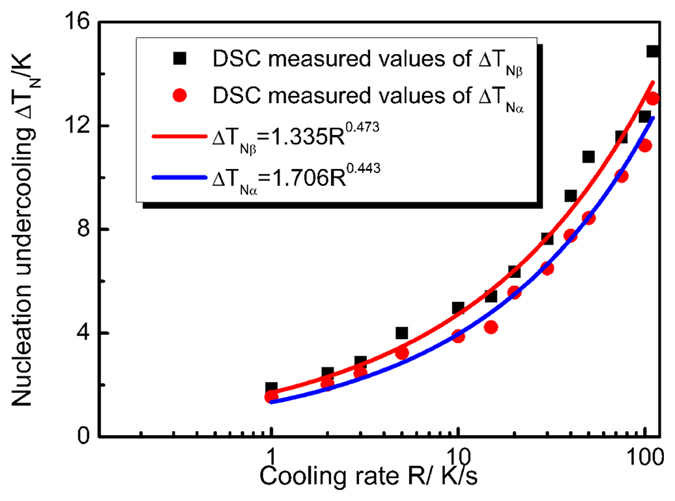
The variation of the measured nucleation undercoolings of both primary Ni_3_Sn_2_ and peritectic Ni_3_Sn_4_ phases with cooling rate.

Equation (7) and (8) show that the values of the nucleation undercooling are closely associated with band spacing. To describe the influence of thermosolutal convection on wavelength, the extent of convection is characterized by comparing the wavelength obtained in diffusive/convective cases, and the nucleation undercooling of the two phases are needed. Figure 7 shows the measured nucleation undercoolings of both primary and peritectic phases. Statistical regression analysis shows the relations between nucleation undercoolings and cooling rate *R*:


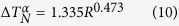



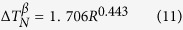


**Figure 8:**
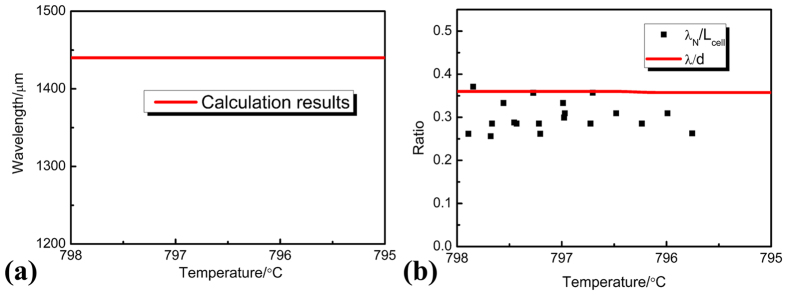
The effect of convection on the morphology of this oscillatory microstructure. (**a**) the calculation on the wavelength in the purely diffusive condition, (**b**) the comparison between these two ratios: the ratio of the wavelength of this oscillatory microstructure to the width of the cells *λ*_*N*_/*L*_*cell*_, and the ratio of the wavelength obtained under diffusive condition to the diameter of the sample *λ*/*d*.

The regression coefficient of these two fit are *r*^2^ = 0.990, 0.984 respectively. In the present work, the cooling rate *R* is 0.04 K/s. As this cooling rate is too low for the Differential Scanning Calorimetry (DSC) measurement, thus, according to Equation (10) and (11), 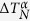 and 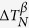
are 0.291 and 0.410 K. As the nucleation undercooling will not be influenced by convection^10^, the above values are used in the following calculation. Through Equations (4, the wavelength of this oscillatory microstructure in the case of diffusive condition is obtained. The results in Fig. 8a show the wavelengths predicted by the diffusive model^9^. In order to eliminate the influence of peritectic transformation on wavelength, only the results in the vicinity of T_P_ is illustrated. It can be found from Fig. 8b that the diffusive predictions in Fig. 8a are much larger than the experimental results. Here *λ*_*N*_ is the wavelength of this oscillatory microstructure, *L*_*cell*_ is the width of the cells, *λ* is the wavelength obtained under diffusive condition, and *d* is the diameter of the sample. Compared with the diffusive predictions, the change in wavelengths is especially not obvious in the present work, which indicates that the oscillatory thermosolutal convection is weak in the present work. This further confirms the conclusion above that the thermosolutal convection is not heavy in this work, that is, 10 < *U*_*C*_/*v*_*P*_ < 100.

But it should be noted that the environment where this oscillatory microstructure evolves is distinct from that of the previous researches^8^,^9^,^10^,^12^. The much smaller dimension of this oscillatory microstructure is due to the smaller cell spacing of Sn–36at.% peritectic alloy^38^. As shown in Equations (10) and (11), the nucleation undercoolings of Ni_3_Sn_2_/Ni_3_Sn_4_ phases are really small. In addition, the cell spacing of Ni_3_Sn_2_ phase is smaller, and the experimental results show that the widths of cells *L*_*cell*_ are always smaller than 100 μm in the present work. As a result, it can be concluded that the nucleation rate of Ni_3_Sn_2_ phase is not slow when the nucleation undercooling required for Ni_3_Sn_2_ phase is satisfied. Therefore, Thus, the convection filed whose dimension was analogous to the diameter of the sample^8^,^9^,^10^,^12^ is divided into many local fields which are limited by the cells. Although these local convection fields are interconnected with each other, the oscillatory thermosolutal convection has been limited to local positions to a large extent. This can be confirmed by the results in Fig. 8b. The ratio of the wavelength of this oscillatory microstructure to the width of the cells *λ*_*N*_/*L*_*cell*_ is quite close to the ratio of the wavelength obtained under diffusive condition to the diameter of the sample *λ*/*d*. Besides, the small change in *λ*_*N*_/*L*_*cell*_ from about 0.36 to 0.26 confirms the weak oscillatory thermosolutal convection in the present work.

In addition, it can be deduced from Fig. 8b that at temperatures close to T_P_ where peritectic reaction occurs, the thermosolutal convection is more instable. This is in consistent with what we have discussed in the experiments. Due to the continuous rejection of Sn into the melt at T_P_, oscillatory thermosolutal convection is more easily formed. As a result, this oscillatory microstructure can not be observed at the bottom of the cells. This is because that the oscillatory thermosolutal convection in the mushy zone is sensitive to the local morphology of structures. At the bottom of the cells, the permeability is lower than that at the middle of the cells, leading to relatively weaker oscillatory thermosolutal convection at the bottom of the cells. Therefore, this oscillatory microstructure is less likely formed at the bottom of the cells.

As has been discussed above, this oscillatory microstructure is formed due to oscillatory convection. The requirement of oscillatory convection can not be easily satisfied in peritectic systems where both primary and peritectic phases are solid solution phase. During solidification of solid solution phase, a time-independent steady state solute redistribution can be established. Thus, oscillatory convection can not be easily satisfied while the solute redistribution in the liquid at the growing interface can not reach the steady state for intermetallic compound with nil solubility unless the initial composition of the alloy equals to the composition of the intermetallic compound^24^. For intermetallic compounds with narrow solubility range^39^, since the initial composition of the alloy can not equal to the composition of the intermetallic compound with narrow solubility range, the melt concentration at the interface decreases following the liquidus line of primary α phase. If the undercooling required for nucleation of the peritectic β phase is satisfied, it is possible to nucleate β phase.

Thus, in this work, as Sn atoms are continuously rejected into the melt during solidification, Sn concentration continues increasing in the melt. This non-steady state solute redistribution is suitable for the formation of oscillatory convection, leading to formation of this oscillatory microstructure. Therefore, the solidification characteristics of peritectic systems containing intermetallic compounds with narrow solubility are distinct from both solid solution phases and intermetallic compounds with nil solubility. Due to their small ranges of solubility, peritectic transformation can take place during peritectic solidification; due to non-steady state solute redistribution of them, oscillatory convection can be more easily satisfied. So, this kind of peritectic systems can not be simply regarded as the intermediate state between those containing solid solution phases and those containing intermetallic compounds with nil solubility range.

## Conclusion

An oscillatory microstructure has been observed for the first time during deep-cellular growth of directionally solidified Sn–36at.%Ni alloy where both the primary and peritectic phases are intermetallic compounds with narrow solubility. The initial morphology of this wave-like oscillatory structure is caused by oscillatory convection of the mushy zone during solidification. And its dimension is much smaller as compared with those observed in previous studies, which is associated with smaller cell spacing of this alloy. The influence of convection on this oscillatory microstructure has been characterized through its “wavelength”. Due to peritectic transformation, morphology of this oscillatory structure changes during following solidification. It has also been found that, due to the solute redistribution during solidification of intermetallic compounds with narrow solubility range, the oscillatory convection can be more easily formed in this alloy.

## Methods

Sn–36at.%Ni peritectic alloy was induction melted under an argon atmosphere from pure Ni and Sn (99.9%). As-cast rods of 3 mm in diameter and 110 mm in length were cut from the ingot. The experiments consisting of melting followed by directional solidification were carried out in a Bridgman-type system consisting of a resistance furnace, a water cooled liquid metal bath filled with liquid Ga–In–Sn alloy, which has been described^4^,^5^,^6^,^7^,^8^. Temperature profiles were measured separately using a PtRh30-PtRh6 thermocouple inserted within a fine alumina tube (0.6 mm in inner diameter) down to the center of the samples. During the pulling process, the thermocouple moved downwards with the sample at the same pulling rate. The temperature gradient close to the solid/liquid interface was deduced from the temperature profiles and was approximately 40 K/mm.

Solidification of Sn–36at.%Ni peritectic alloy was carried out at growth rate of 1 μm/s in a constant temperature gradient (40 K/mm). With a predetermined growth distance of 30 mm reached, the samples were quenched into liquid Ga-In-Sn alloy quickly to preserve the microstructure. Then, the samples were longitudinally sectioned, polished and etched with a solution of 10 g FeCl_3_−20 ml HCl-180 ml H_2_O for further analysis. The microstructures of the longitudinal section of rods was analyzed by scanning electron microscopy (SEM (Quanta-200)). Energy dispersive X-ray spectrometer (EDS) was applied to determine composition of phases.

The measurement on the nucleation undercooling of both primary Ni_3_Sn_2_ and peritectic Ni_3_Sn_4_ phases were performed by differential scanning clorimetry (DSC-SETARAM) in a continuous mode. The calorimeter was calibrated by measuring the melting temperatures (T_M_) of metallic In, Sn, Al, Au and Pd (99.999 mass % purity). T_M_ was obtained with an accuracy of T_M_ ± 0.5 °C for all cases. As-cast rods of 8 mm in diameter and length of 10 mm were put into a high-purity alumina crucible of 20 mm length and 10 mm inner diameter. After heating to 1,250 °C at a rate of 10 °C/min, the sample was held for 30 min at this temperature, then the samples were cooled to room temperatureat a range of cooling rates 1 ~ 100 °C/s. All the experiments were carried out in Ar atmosphere.

## Additional Information

**How to cite this article**: Peng, P. *et al.* On oscillatory microstructure during cellular growth of directionally solidified Sn–36at.%Ni peritectic alloy. *Sci. Rep.* **6**, 24315; doi: 10.1038/srep24315 (2016).

## References

[b1] Kerr H. & Kurz W. Solidification of peritectic alloys. Inter. Mater. Rev. 41, 129–164 (1996).

[b2] Umeda T., Okane T. & Kurz W. Phase selection during solidification of peritectic alloys. Acta Mater. 44, 4209–4216 (1996).

[b3] Kohler F., Germond L., Wagnière J. D. & Rappaz M. Peritectic solidification of Cu–Sn alloys: Microstructural competition at low speed. Acta Mater. 57, 56–68 (2009).

[b4] Peng P. *et al.* Effect of peritectic reaction on dendrite coarsening in directionally solidified Sn-36at.%Ni alloy. J. Mater. Sci. 47, 6108–6117 (2012).

[b5] Liu D. M. *et al.* Secondary dendrite arm migration caused by temperature gradient zone melting during peritectic solidification. Acta Mater. 60, 2679–2688 (2012).

[b6] Li X. Z. *et al.* Dendrite coarsening in directionally solidified Sn–36at.%Ni peritectic alloy in the presence of the migration of secondary dendrite arms. Mater. Chem. Phys. 145, 203–212 (2014).

[b7] Hu X. W., Li S. M., Gao S. F., Liu L. & Fu H. Z. Peritectic transformation and primary α-dendrite dissolution in directionally solidified Pb–26%Bi alloy. J. Alloy Compds. 501, 110–116 (2010).

[b8] Trivedi R. & Park J. S. Dynamics of microstructure formation in the two-phase region of peritectic systems. J. Cryst. Growth 235, 572–588 (2002).

[b9] Trivedi R. Theory of layered-structure formation in peritectic systems, Metall. Mater. Trans. A 26A, 1583–1590 (1995).

[b10] Mazumder P., Trivedi R. & Karma A. A model of convection-induced oscillatory structure formation in peritectic alloys. Metall. Mater. Trans. A 31A, 1233–1246 (2000).

[b11] Trivedi R. The role of heterogeneous nucleation on microstructure evolution in peritectic systems. Scripta Mater. 53, 47–52 (2005).

[b12] Park J. S. & Trivedi R. Convection-induced novel oscillating microstructure formation in peritectic systems. J. Cryst. Growth 187, 511–515 (1998).

[b13] Lo T. S., Karma A. & Plapp M. Phase-field modeling of microstructural pattern formation during directional solidification of peritectic alloys without morphological instability. Phys. Rev. E 63, 031504-1–031504-15 (2003). 10.1103/PhysRevE.63.031504 11308654

[b14] Boettinger W. J. The structure of directionally solidified two-phase Sn-Cd peritectic alloys. Metall. Trans. 5, 2023–2031 (1974).

[b15] Karma A., Rappel W. J., Fuh B. C. & Trivedi R. Model of banding in diffusive and convective regimes during directional solidification of peritectic systems, Metall. Mater. Trans. A 29A, 1457–1470 (1998).

[b16] Liu S. & Trivedi R. Effect of thermosolutal convection on microstructure formation in the Pb-Bi peritectic system. Metall. Mater. Trans. A 37A, 3293–3304 (2006).

[b17] Wang L. *et al.* The effect of the flow driven by a travelling magnetic field on solidification structure of Sn–Cd peritectic alloys. J. Cryst. Growth 356, 26–32 (2012).

[b18] Luo W. Z., Shen J., Min Z. X. & Fu H. Z. A band microstructure in directionally solidified hypo-peritectic Ti–45Al alloy. Mater. Lett. 63, 1419–1421 (2009).

[b19] Li X. *et al.* Effect of a high magnetic field on the morphological instability and irregularity of the interface of a binary alloy during directional solidification. Acta Mater. 57, 1689–1701 (2009).

[b20] Li X. *et al.* Effect of a high magnetic field on the microstructures in directionally solidified Zn–Cu peritectic alloys. Acta Mater. 73, 83–96 (2014).

[b21] Wang L. S., Shen J., Feng Z. R. & Fu H. Z. Effect of rotating magnetic field on microstructure formation of directionally solidified Sn–1.6Cd peritectic alloy. Appl. Phys. A 113, 177–183 (2013).

[b22] Wang L. S., Shen J., Wang L., Du Y. J. & Fu H. Z. Formation mechanism of banded structure during directional solidification of Sn–Cd peritectic alloy under convection condition. Appl. Phys. A 114, 769–776 (2014).

[b23] Wang L. *et al.* Influences of travelling magnetic field on the dendritic structures of Sn–1.8Cd peritectic alloy during directional solidification. Appl. Phys. A 112, 363–370 (2013).

[b24] Liu D. M. *et al.* Solute redistribution during planar growth of intermetallic compound with nil solubility. Intermetallics 26, 131–135 (2012).

[b25] Trivedi R. & Kurz W. Modeling of solidification microstructures in concentrated solutions and intermetallic systems. Metall. Mater. Trans. A 21A, 1311–1318 (1990).

[b26] Peng P. *et al.* Microstructural length scales in directionally solidified Sn-40at.%Mn peritectic alloy containing intermetallic compounds. Intermetallics 55, 73–79 (2014).

[b27] Duan G. H., Liu Y. C., Yang G. C. & Zhou Y. H. Morphological evolution of banding structures at high solidification velocity. Mater. Lett. 57, 1091–1095 (2003).

[b28] Liu Y. C., Yang G. C. & Zhou Y. H. High-velocity banding structure in the laser-resolidified hypoperitectic Ti47Al53 alloy. J. Cryst. Growth 240, 603–610 (2002).

[b29] Carrard M., Gremaud M., Zimmermann M. & Kurz W. About the banded structure in rapidly solidified dendritic and eutectic alloys. Acta Metall. Mater. 40, 983–996 (1992).

[b30] Schmetterer C. *et al.* Intermetallics 15, 869–884 (2007).

[b31] Akamatsu S., Moulinet S. & Faivre G. The formation of lamellar-eutectic grains in thin samples. Metall. Mater. Trans. A 32A, 2039–2048 (2001).

[b32] Gandin Ch-A., Eshelman M. & Trivedi R. Orientation dependence of primary dendrite spacing. Metall. Mater. Trans. A 27A, 2727–2739 (1996).

[b33] St John D. H. & Hogan L. M. A simple prediction of the rate of the peritectic transformation. Acta Metall. 35, 171–174 (1987).

[b34] Das A., Manna I. & Pabi S. K. A numerical model of peritectic transformation. Acta Mater. 47, 1379–1388 (1999).

[b35] Ha H. P. & Hunt J. D. A numerical and experimental study of the rate of transformation in three directionally grown peritectic systems. Metall. Mater. Trans. A 31A, 29–34 (2000).

[b36] Gur D. & Bamberger M. Reactive isothermal solidification in the Ni–Sn system. Acta Mater. 46, 4917–4923 (1998).

[b37] Shen J., Chan Y. C. & Liu S. Y. Growth mechanism of Ni3Sn4 in a Sn/Ni liquid/solid interfacial reaction. Acta Mater. 57, 5196–5206 (2009).

[b38] Tiller W. A., Jackson K. A., Rutter J. W. & Chalmers B. The redistribution of solute atoms during the solidification of metals. Acta Metall. 1, 428–437 (1953).

[b39] Peng P. *et al.* Composition-dependent phase substitution in directionally solidified Sn-22at.%Ni peritectic alloy. J Mater. Sci. 55, doi: 10.1007/s10853-015-9472-4 (2015)

